# Supplementary material for: Race, Ethnicity, and Geography as Determinants of Excessive Weight and Low Physical Activity in Pediatric Population: Protocol for Systematic Review and Meta-Analysis
Source: Healthcare (Basel). 2024 Sep 13;12(18):1830. doi: 10.3390/healthcare12181830 (PMC11431668; doi:10.3390/healthcare12181830)
Supplement: Supplementary file 1 [file healthcare-12-01830-s001.zip › healthcare-3147528-supplementary/healthcare-3147528-supplementary table S2-9.6.pdf]

Supplementary Table S1. Characteristics of studies included in pilot meta-analysis

First author and publication year	Country	Race/ethnicity	Sample size	Age of participants
Bernhardsen et al., 2019[1]	Norway	N/A	Boys: 242; Girls: 203.	9 to 12 years at the time of follow-up
Ferrari et al., 2015 [2]	Brazil	N/A	Boys: 238; Gils: 247.	9 to 11 years
Gába et al, 2017[3]	Czech Republic	N/A	Boys: 156; Girls: 209.	7 to 12 years
Janz et al., 2017 [4]	Canada	Predominately white	Boys: 230; Girls: 233.	Data collected in several measurement waves at approximately ages 5, 8, 11, 13, 15, 17 and 19
Diouf et al., 2016 [5]	Senegal	N/A	Boys: 20; Girls: 22.	8 to 11 years
Anderson et al., 2017 [6]	New Zealand	Māori: 45%; New Zealand European: 45%; other ethnicities: 10%.	239 children; Girls: 52%	4.8 to 16.8 years
Dalene et al., 2017 [7]	Norway	N/A	5825 participants	6-, 9- and 15-year-olds
Byrd-Williams, et al., 2007 [8]	USA	Hispanic: 63%; Non-Hispanic:	169 children; Girls: 49.5%	9.4±0.4*
Steele et al., 2009 [9]	UK	Predominately white	Boys: 820; Girls: 1042.	9 to 10 years
Drenowatz et al., 2010 [10]	USA	Study 1: Caucasian: 88.0%; African-American: 3.3%, Hispanic: 1.5%; other: 7.3%.<br><br>Study 2: Caucasian: 93.7%.	Study 1: Boys: 117; Girls: 154.<br><br>Study 2: Boys: 63; Girls: 68.	Study 1: 9.6 ± 0.9*<br><br>Study 2: 8.8 ± 1.7*

Katzmarzyk et al., 2015 [11]	Australia, Brazil, Canada, China, Colombia, Finland, India, Kenya, Portugal, South Africa, UK, and USA	N/A	6539 children	9 to 11 years
Katzmarzyk, 2008 [12]	Canada	Aboriginal Canadians: 4,98%; Non-aboriginal Canadians: 95,02%	Boys: 6320; Girls: 6085.	2 to 17 years
Hodgkin et al., 2010 [13]	New Zealand	European: 30.3%; Maori: 37.3%; Pacific: 32.4%.	Boys: 1697; Girls: 1578.	5 to 15 years
Gordon-Larsen et al., 2002 [14]	USA	non-Hispanic white: 54.8%; non-Hispanic black: 20.9%; Hispanic: 17.1%; Asian: 7.2%.	Boys: 49.3% Girls:50.7%.	11 to 19 years
Belcher et al., 2010 [15]	USA	non-Hispanic White: 69.9%; non-Hispanic Black: 16.6%; Mexican American: 13.5%.	Boys: 1659; Girls: 1537	6 to 19 years
Mitchell et al., 2013 [16]	USA	White: 81.7%; Other: 18.3%.	Boys: 461; Girls: 477.	Data collected in several measurement waves at approximately ages 9, 11, 12, and 15
Labree et al., 2015 [17]	The Netherlands	native Dutch: 79.6%; Turkish: 4.8%; Moroccan: 3.4%;	Boys: 970; Girls: 973.	8 to 9 years

		other non-western: 5.4%; other western: 6.8%.		
McMurray et al., 2000 [18]	USA	White: 77%; African American: 23%	Boys: 1149; Girls: 1240.	10 to 16 years
Gordon-Larsen et al., 1999 [19]	USA	White: 54.8%; Black: 21.5%; Hispanic: 17.2%; Asian: 6.5%.	Boys: 6456; Girls: 6701.	12 to 22 years
Laguna et al., 2013 [20]	Spain	N/A	Boys: 374; Girls: 387.	9- and 15-year-olds
Gutin et al., 2005 [21]	USA	Black: 51.1%; White: 48.9%.	Boys: 196; Girls: 225.	16.2±1.2*
Moliner-Urdiales et al. 2009 [22]	Spain	N/A	Boys: 183; Girls: 182.	12.5 to 17.5 years
Martinez-Gomez et al., 2010 [23]	Greece, Germany, Belgium, France, Hungary, Italy, Sweden, Austria, Spain.	N/A	Boys: 973; Girls: 1121.	12.5 to 17.5 years
Silva et al., 2010 [24]	Portugal, Spain.	N/A	Portugal: Boys: 43%; Girls:57%; Total: 105<br><br>Spain: Boys: 60%; Girls: 40%. Total: 95.	Portugal: 14.56±1.50*<br><br>Spain: 13.66±1.15*
Lohman et al., 2008 [25]	USA	Hispanic: 21%; non-Hispanic Black: 21%; non-Hispanic white: 45%; non-Hispanic other: 13%.	Boys: N/A; Girls: 1440.	14.1±0.54*
van Stralen et al., 2014 [26]	Belgium, Greece,	N/A	Boys: 49%; Girls: 51%.	10 to 12 years

	Hungary, Netherlands, Switzerland		Total: 1025.	
Dencker et al., 2006 [27]	Sweden	N/A	Boys: 140; Girls: 108.	7.9 to 11.1 years
Ruh et al. 2003 [28]	New Zealand	European: 31.9%; Maori: 34.3%; Pacific: 33.8%.	Boys: 83; Girls: 89.	5 to 14 years
Richardson et al. 2014 [29]	USA	European Americans: 62.5%; African–Americans: 21.4%; Hispanic Americans: 16.1%.	Boys: 47.1%; Girls: 52.9%; Total: 8113	11 to 22 years 16.4±1.8*
Al-Hazzaa el al., 2012 [30]	Saudi Arabia	N/A	Boys: 1400; Girls: 1506.	14 to 19 years
Collison et al., 2010 [31]	Saudi Arabia	N/A	Boys: 5033; Girls: 4400.	14 to 19 years
Schneider et al., 2007[32]	USA	European-American: 60%; Asian: 14%; Latina: 19%; mixed or other: 7%.	Boys: N/A; Girls: 194.	14 to 17 years
Hanson et al., 2007 [33]	USA	Caucasian: 42%; African American: 56%; Others: 2%.	Boys: 39%; Girls: 61%; Total: 113.	16 to 19 years
Merrigan et al., 2021 [34]	USA	Latino	Boys: 16; Girls: 7.	7.9 ± 1.4*
Klein_Platat et al., 2005 [35]	France	N/A	Boys: 1357; Girls: 1357.	12 years
Micklesfield et al., 2014 [36]	South Africa	N/A	Sample 1: Boys: 98; Girls: 97. Sample 2: Boys: 91; Girls: 95.	Sample 1: 11 to 12 years<br><br>Sample 2: 14 to 15 years.
Al-Hazzaa et al., 2011 [37]	Saudi Arabia	N/A	Boys: 39; Girls: 36.	16.1±1.1*
Tayyem et al., 2012 [38]	Jordan	N/A	Boys: 386; Girls: 349.	14 to 18 years

Wang et al., 2016 [39]	Hong Kong	Chinese	Boys: 412;<br>Girls: 330.	8 to 13 years
Duncan et al., 2014 [40]	Saudi Arabia and Britain	N/A	Boys: 1382;<br>Girls: 1417.	14 to 18 years
Al-Hazzaa et al., 2011 [41]	Saudi Arabia	N/A	Boys: 1401;<br>Girls: 1507.	14 to 19 years
Allafi et al., 2014 [42]	Kuwait	N/A	Boys: 463;<br>Girls: 443.	14 to 19 years
Lee et al., 2016 [43]	USA	Native American: 5.7%;<br>Asian: 1.1%;<br>African American: 2.7%;<br>White: 73.6%;<br>Other: 16.9%.	Boys: 127 ;<br>Girls: 134.	7 to 9 years
Larouche et al., 2019 [44]	Kenya<br>South Africa<br>Brazil<br>Colombia<br>Canada<br>USA<br>Finland<br>Portugal<br>UK<br>India<br>China<br>Australia	N/A	Boys: 2956;<br>Girls: 3522.	9 to 11 years
Naseri et al., 2020 [45]	Iran	N/A	Boys: 523;<br>Girls: 700.	12 to 18 years
Farooq et al., 2021 [46]	UK	N/A	7-years-old:<br>Boys: 279;<br>Girls: 283.<br><br>9-years-old:<br>Boys: 277;<br>Girls: 287.<br><br>12-years-old:<br>Boys: 246;<br>Girls: 260.<br><br>15-years-old:<br>Boys: 173;<br>Girls: 155.	Data collected in several measurement waves at approximately ages 7, 9, 12, and 15
Pate et al., 2019 [47]	USA	Black: 36.2%;<br>White: 37.9%;	Boys: 297;<br>Girls: 355.	10 to 12 years at baseline

		Hispanic: 9.2%; Other: 16.7%.		
Farooq et al., 2018 [48]	UK		7-years-old: Boys: 217; Girls: 214.<br><br>9-years-old: Boys: 209; Girls: 219.<br><br>12-years-old: Boys: 176; Girls: 209.<br><br>15-years-old: Boys: 131; Girls: 147.	Data collected in several measurement waves at approximately ages 7, 9, 12 and 15 years
Basterfield et al. 2011	UK	N/A	Boys: 198; Girls: 207.	7 years at baseline
Aparicio-Ugarriza et al. 2020 [49]	Spain	N/A	Sample 1: Boys: 126; Girls: 87.<br><br>Sample 2: Boys: 137; Girls: 74.	Sample 1: 9 to 12 years<br><br>Sample 2: 13 to 17 years
Werneck et al., 2020 [50]	UK, Denmark, Estonia, Portugal, United States of America	N/A	Boys: 2486; Girls: 2730.	Assessment at approximately 24 years
Li et al., 2019 [51]	Australia, Brazil, Canada, China, Colombia, Finland, India, Kenya, Portugal, South Africa, UK, US.	N/A	Boys: 2601; Girls: 3178.	9 to 11 years

Wong et al., 2015 [52]	Hong Kong	Chinese	Boys: 52.6%; Girls: 47.4%; Total: 1666.	6 to 8 years
Fairclough et al., [53]	UK	N/A	Boys: 78; Girls: 97.	10 to 11 years
Machado-Rodrigues, et al., [54]	Portugal	N/A	Boys: 165; Girls: N/A.	13 to 16 years
Basterfield et al., 2015 [55]	UK	N/A	7-years-old: Boys: 302; Girls: 307.<br><br>9-years-old: Boys: 292; Girls: 293.<br><br>12-years-old: Boys: 257; Girls: 268.	Data collected in several measurement waves at approximately ages 7, 9 and 12 years
Ferrari et al., 2015 [56]	Brazil		Boys: 216; Girls: 225.	10 years
Steele et al., 2010 [57]	UK	Predominately white	Boys: 701; Girls: 867.	9 to 10 years
Mitchell et al., 2013 [58]	USA	N/A	9-years-old: Total: 740.<br>11-years-old: Total: 681.<br><br>12-years-old: Total: 540.<br><br>15-years-old: Total: 424.	Data collected in several measurement waves at approximately ages 9, 11, 12 and 15 years
Keane et al., 2017 [59]	Ireland	N/A	Boys: 464; Girls: 362.	8 to 11 years
Goldfield et al., 2007 [60]	Canada	N/A	Boys: 13; Girls: 17.	8 to 12 years
Epstein et al., 2007 [60]	USA	White :87.9%; Black: 6.9%; Hispanic: 3.4%; other: 1.7%.	Boys: 28; Girls: 30.	8 to 16 years
Saunders et al., 2013 [61]	Canada	N/A	Boys: 286; Girls: 236.	8 to 11 years

Gaya et al., 2009 [62]	Portugal	N/A	Boys: 66; Girls: 97.	11 to 17 years
Ochoa et al., 2007 [63]	Spain	N/A	Total: 370.	6 to 18 years
Treuth et al., 2009 [64]	USA	N/A	Girls: 984 in each sample. No boys.	Sample 1: 11.9±0.4* Sample 2: 13.9±0.4*
Nogueira et al., 2009 [65]	Brazil	N/A	Boys: 204; Girls: 122.	11 to 15 years
Sardinha et al., 2008 [66]	Portugal		Boys: 161; Girls: 147.	9 to 10 years
Epstein et al., 2008 [67]	USA	N/A	Total: 70 children.	4 to 7 years
Gortmaker et al., 1999 [68]	USA	Predominately white (over 60%).	Girls: 627; Boys: 668.	Approximately 11 years old students
Robinson, T., 1999 [69]	USA		Total: 192	Approximately 8 years old students
Burke et al., 2006 [70]	Australia	N/A	Boys: 281; Girls: 321.	11 to 14 years
Berkey et al., 2003 [71]	USA	N/A	Boys: 5120; Girls: 6767.	10 to 15 years
Fulton et al., 2009 [72]	USA	N/A	Boys: 227; Girls: 245.	Children were enrolled at 8, 11 and 14 years and followed-up for 4 years.
Berkey et al., 2000 [73]	USA	N/A	Boys: 4620; Girls: 6149.	9 to 14 years
Steffen et al., 2009 [74]	USA	Sample 1: Black: 12%; Nonblack: 88%. Sample 2: Black: 12%; Nonblack: 88%. Sample 2: Black: 7%; Nonblack: 93%.	Sample 1: Boys: 118; Girls: 118. Sample 2: Boys: 75; Girls: 82. Sample 3: Boys: 63; Girls: 70.	Sample 1: 8.1±0.2* Sample 2: 11.1±0.3* Sample 3: 14.0±0.1*
Pratt et al., 2008 [75]	USA	Caucasians: 44.3%; African Americans: 22%;	Boys: N/A; Girls:1458.	12 years old

		Hispanics: 22%; Asians: 3.7%; American Indians: 0.8%; Multiracial: 7.5%.		
Laurson et al., 2008 [76]	USA	N/A	Boys: 318; Girls: 391.	7 to 12 years
Butte et al., 2007 [77]	USA	Hispanic	Boys: 441; Girls: 456.	2 to 19 years
Ortega et al., 2007 [78]	Spain	N/A	Boys: 1357; Girls: 1502.	13 to 18.5 years
Obarzanek et al., 1994 [79]	USA	Black: 50.9%; White: 49.1%.	Boys: N/A; Girls: 2379.	9 to 10 years
Katzmarzyk et al., 1998 [80]	Canada	N/A	Boys: 423; Girls: 361.	9 to 18 years
Goldfield et al., 2006 [81]	Canada	N/A	Boys: 13; Girls: 17.	8 to 12 years
Oliveira et al., 2017 [82]	Brazil	White: 66.8%; Black: 7%; Brown: 13.8%; Other: 3.4%; Non-reported: 9%.	Boys: 242; Girls: 252.	10 years
Johansson et al., 2015 [83]	Sweden	N/A	Boys: 61; Girls: 62.	2.03±0.1*
Riddoch et al., 2007 [84]	UK	N/A	Boys: 2662; Girls: 2933.	11 years old
Collings et al., 2017 [85]	UK	South Asian: 55.3%; White British: 36.6%; Other/Mixed: 8.1%	Boys: 169; Girls: 164.	11 months to 5 years
Tambalis et al., 2019 [86]	Greece	N/A	Boys: 87803; Girls: 89288.	8 to 17 years
van Sluijs et al., 2011 [87]	UK	N/A	Boys: 841; Girls: 1067.	10 years old
Hendersen et al., 2012 [88]	Canada	White	Boys: 222; Girls: 202.	8 to 10 years at baseline
Al-Hazzaa et al., 2007 [89]	Saudi Arabia	N/A	Boys: 109; Girls: 115.	5.19 ± 0.85*
Leppanen et al., 2016 [90]	Sweden	N/A	Boys: 170; Girls: 137.	4 years old
Collings et al. 2014 [91]	UK	White: 94.6%; Other: 5.4%.	Boys: 359; Girls: 466.	15.0±0.31*

Wang et al., 2013 [92]	China	N/A	Boys: 1603; Girls: 1518.	9 to 17 years
McVeigh et al. 2004 [93]	South Africa	White: 82; Black: 450.	Boys: 202; Girls: 184.	9 years old
Ojiambo et al., 2012 [94]	Kenya	N/A	Boys: 99; Girls: 101.	12 to 16 years
Prista et al., 2009 [95]	Mozambique	N/A	Boys: 139; Girls: 117.	6 to 16 years
Ni Mhurchu et al., 2008 [96]	New Zealand	N/A	Boys: 12; Girls: 8.	10 to 14 years
Taylor et al., 2011 [97]	New Zealand	N/A	Boys: 235; Girls: 206.	Approximately 8 years
Hume et al., 2008 [98]	Australia	N/A	Boys: 123; Girls: 125.	9 to 12 years
Maddison et al., [99]	New Zealand	Maori: 16.9%; Non-Maori: 83.1%.	Boys: 391; Girls: 325.	10 to 18 years
Fraysse et al., 2019 [100]	Australia		Boys: 632; Girls: 629.	11 to 12 years

*data provided in mean±SD

References:

1. Bernhardsen, G. P. et al. Early life risk factors for childhood obesity—Does physical activity modify the associations? The MoBa cohort study. Scand J Med Sci Sports 29, 1636–1646 (2019).
2. de Moraes Ferrari, G. L. et al. Moderate-to-Vigorous Physical Activity and Sedentary Behavior: Independent Associations With Body Composition Variables in Brazilian Children. Pediatr Exerc Sci 27, 380–389 (2015).
3. Gába, A., Mitáš, J. & Jakubec, L. Associations between accelerometer-measured physical activity and body fatness in school-aged children. Environ Health Prev Med 22, 43 (2017).
4. JANZ, K. F. et al. Physical Activity, Not Sedentary Time, Predicts Dual-Energy X-ray Absorptiometry–measured Adiposity Age 5 to 19 Years. Med Sci Sports Exerc 49, 2071–2077 (2017).
5. Diouf, A. et al. Physical Activity Level and Sedentary Behaviors among Public School Children in Dakar (Senegal) Measured by PAQ-C and Accelerometer: Preliminary Results. Int J Environ Res Public Health 13, 998 (2016).
6. Anderson, Y. C. et al. Physical activity is low in obese New Zealand children and adolescents. Sci Rep 7, 41822 (2017).
7. Dalene, K. E. et al. Cross-sectional and prospective associations between physical activity, body mass index and waist circumference in children and adolescents. Obes Sci Pract 3, 249–257 (2017).
8. Byrd-Williams, C., Kelly, L. A., Davis, J. N., Spruijt-Metz, D. & Goran, M. I. Influence of gender, BMI and Hispanic ethnicity on physical activity in children. International Journal of Pediatric Obesity 2, 159–166 (2007).

9. Steele, R. M., van Sluijs, E. M., Cassidy, A., Griffin, S. J. & Ekelund, U. Targeting sedentary time or moderate- and vigorous-intensity activity: independent relations with adiposity in a population-based sample of 10-y-old British children. Am J Clin Nutr 90, 1185–1192 (2009).
10. Drenowatz, C. et al. Influence of socio-economic status on habitual physical activity and sedentary behavior in 8- to 11-year old children. BMC Public Health 10, 214 (2010).
11. KATZMARZYK, P. T. et al. Physical Activity, Sedentary Time, and Obesity in an International Sample of Children. Med Sci Sports Exerc 47, 2062–2069 (2015).
12. Katzmarzyk, P. T. Obesity and Physical Activity Among Aboriginal Canadians. Obesity 16, 184–190 (2008).
13. Hodgkin, E., Hamlin, M., Ross, J. & Peters, F. Obesity, Energy Intake and Physical Activity in Rural and Urban New Zealand Children. http://www.rrh.org.au.
14. Gordon-Larsen, P., Adair, L. S. & Popkin, B. M. Ethnic Differences in Physical Activity and Inactivity Patterns and Overweight Status. Obes Res 10, 141–149 (2002).
15. BELCHER, B. R. et al. Physical Activity in US Youth. Med Sci Sports Exerc 42, 2211–2221 (2010).
16. Mitchell, J. A. et al. Moderate-To-vigorous physical activity is associated with decreases in body mass index from ages 9 to 15 years. Obesity 21, (2013).
17. Labree, W. et al. Differences in Overweight and Obesity among Children from Migrant and Native Origin: The Role of Physical Activity, Dietary Intake, and Sleep Duration. PLoS One 10, e0123672 (2015).
18. McMurray, R. G. et al. The Influence of Physical Activity, Socioeconomic Status, and Ethnicity on the Weight Status of Adolescents. Obes Res 8, 130–139 (2000).
19. Gordon-Larsen, P., McMurray, R. G. & Popkin, B. M. Adolescent physical activity and inactivity vary by ethnicity: The National Longitudinal Study of Adolescent Health. J Pediatr 135, 301–306 (1999).
20. Laguna, M. et al. Obesity and physical activity patterns in children and adolescents. J Paediatr Child Health 49, 942–949 (2013).
21. Gutin, B., Yin, Z., Humphries, M. C. & Barbeau, P. Relations of moderate and vigorous physical activity to fitness and fatness in adolescents. Am J Clin Nutr 81, 746–750 (2005).
22. Moliner-Urdiales, D. et al. Association of objectively assessed physical activity with total and central body fat in Spanish adolescents; The HELENA Study. Int J Obes 33, 1126–1135 (2009).
23. Martinez-Gomez, D. et al. Recommended Levels of Physical Activity to Avoid an Excess of Body Fat in European Adolescents. Am J Prev Med 39, 203–211 (2010).
24. Silva, P. et al. Differences in the physical activity pattern between Portuguese and Spanish adolescents. Archives of Exercise in Health and Disease 1, 26–31 (2010).
25. LOHMAN, T. G. et al. Relationships among Fitness, Body Composition, and Physical Activity. Med Sci Sports Exerc 40, 1163–1170 (2008).

26. van Stralen, M. M. et al. Measured sedentary time and physical activity during the school day of European 10- to 12-year-old children: The ENERGY project. J Sci Med Sport 17, 201–206 (2014).
27. Dencker, M. et al. Daily physical activity and its relation to aerobic fitness in children aged 8–11 years. Eur J Appl Physiol 96, 587–592 (2006).
28. Rush, E. C., Puniani, K., Valencia, M. E., Davies, P. S. W. & Plank, L. D. Estimation of body fatness from body mass index and bioelectrical impedance: comparison of New Zealand European, Maori and Pacific Island children. Eur J Clin Nutr 57, 1394–1401 (2003).
29. Richardson, A. S. et al. Moderate to vigorous physical activity interactions with genetic variants and body mass index in a large US ethnically diverse cohort. Pediatr Obes 9, (2014).
30. Al-Hazzaa, H. M., Abahussain, N. A., Al-Sobayel, H. I., Qahwaji, D. M. & Musaiger, A. O. Lifestyle factors associated with overweight and obesity among Saudi adolescents. BMC Public Health 12, 354 (2012).
31. Collison, K. S. et al. Sugar-sweetened carbonated beverage consumption correlates with BMI, waist circumference, and poor dietary choices in school children. BMC Public Health 10, 234 (2010).
32. Schneider, M., Dunton, G. F. & Cooper, D. M. Media Use and Obesity in Adolescent Females. Obesity 15, 2328–2335 (2007).
33. Hanson, M. D. & Chen, E. Socioeconomic Status, Race, and Body Mass Index: The Mediating Role of Physical Activity and Sedentary Behaviors during Adolescence. J Pediatr Psychol 32, 250–259 (2006).
34. Merrigan, J. J. et al. Bidirectional Associations between Physical Activity and Sleep in Early-Elementary-Age Latino Children with Obesity. Sports 9, 26 (2021).
35. Klein-Platat, C. et al. Physical activity is inversely related to waist circumference in 12-y-old French adolescents. Int J Obes 29, 9–14 (2005).
36. Micklesfield, L. K. et al. Physical activity and sedentary behavior among adolescents in rural South Africa: levels, patterns and correlates. BMC Public Health 14, 40 (2014).
37. Al-Hazzaa, H. M., Al-Sobayel, H. I. & Musaiger, A. O. Convergent Validity of the Arab Teens Lifestyle Study (ATLS) Physical Activity Questionnaire. Int J Environ Res Public Health 8, 3810–3820 (2011).
38. Tayyem, R. F. et al. Dietary Habits and Physical Activity Levels in Jordanian Adolescents Attending Private versus Public Schools العامة املدارس مقابل اخلاصة باملدارس امللتحقني األردنيني املراهقني بني البدين النشاط ومستوى الغذائية العادات. (2014) 20 .vol.
39. Wang, J. J., Baranowski, T., Lau, W. C. P., Chen, T. A. & Pitkethly, A. J. Validation of the Physical Activity Questionnaire for Older Children (PAQ-C) among Chinese Children. Biomedical and Environmental Sciences 29, 177–186 (2016).
40. Duncan, M. J. et al. Anthropometric and lifestyle characteristics of active and inactive saudi and british adolescents. American Journal of Human Biology 26, 635–642 (2014).
41. Al-Hazzaa, H. M., Abahussain, N. A., Al-Sobayel, H. I., Qahwaji, D. M. & Musaiger, A. O. Physical activity, sedentary behaviors and dietary habits among Saudi adolescents relative to age, gender and region. International Journal of Behavioral Nutrition and Physical Activity 8, 140 (2011).

42. Allafi, A. et al. Physical activity, sedentary behaviours and dietary habits among Kuwaiti adolescents: gender differences. Public Health Nutr 17, 2045–2052 (2014).
43. Lee, J. E., Stodden, D. F. & Gao, Z. Young Children's Energy Expenditure and Moderate-to-vigorous Physical Activity on Weekdays and Weekends. J Phys Act Health 13, 1013–1016 (2016).
44. Larouche, R. et al. Relationships Between Outdoor Time, Physical Activity, Sedentary Time, and Body Mass Index in Children: A 12-Country Study. Pediatr Exerc Sci 31, 118–129 (2019).
45. Naseri, P. et al. Longitudinal association between body mass index and physical activity among adolescents with different parental risk: a parallel latent growth curve modeling approach. International Journal of Behavioral Nutrition and Physical Activity 17, 59 (2020).
46. Farooq, A. et al. Moderate-To-Vigorous Intensity Physical Activity and Sedentary Behaviour across Childhood and Adolescence, and Their Combined Relationship with Obesity Risk: A Multi-Trajectory Analysis. Int J Environ Res Public Health 18, 7421 (2021).
47. Pate, R. R., Schenkelberg, M. A., Dowda, M. & McIver, K. L. Group-based physical activity trajectories in children transitioning from elementary to high school. BMC Public Health 19, 323 (2019).
48. Farooq, M. A. et al. Timing of the decline in physical activity in childhood and adolescence: Gateshead Millennium Cohort Study. Br J Sports Med 52, 1002–1006 (2018).
49. Aparicio-Ugarriza, R. et al. Active Commuting, Physical Activity, and Sedentary Behaviors in Children and Adolescents from Spain: Findings from the ANIBES Study. Int J Environ Res Public Health 17, 668 (2020).
50. Werneck, A. O. et al. Physical activity attenuates metabolic risk of adolescents with overweight or obesity: the ICAD multi-country study. Int J Obes 44, 823–829 (2020).
51. Li, N. et al. Joint associations between weekday and weekend physical activity or sedentary time and childhood obesity. Int J Obes 43, 691–700 (2019).
52. Wong, S. H.-S., Huang, W. Y. & He, G. Longitudinal changes in objectively measured physical activity differ for weekdays and weekends among Chinese children in Hong Kong. BMC Public Health 15, 1310 (2015).
53. Fairclough, S. J., Ridgers, N. D. & Welk, G. Correlates of Children's Moderate and Vigorous Physical Activity During Weekdays and Weekends. J Phys Act Health 9, 129–137 (2012).
54. Machado-Rodrigues, A. M. et al. Physical Activity and Energy Expenditure in Adolescent Male Sport Participants and Nonparticipants Aged 13 to 16 Years. J Phys Act Health 9, 626–633 (2012).
55. Basterfield, L. et al. Longitudinal associations between sports participation, body composition and physical activity from childhood to adolescence. J Sci Med Sport 18, 178–182 (2015).
56. Ferrari, G. L. de M., Araújo, T. L., Oliveira, L. C., Matsudo, V. & Fisberg, M. Association between electronic equipment in the bedroom and sedentary lifestyle,

physical activity, and body mass index of children. J Pediatr (Rio J) 91, 574–582 (2015).
57. Steele, R. M. et al. An investigation of patterns of children's sedentary and vigorous physical activity throughout the week. International Journal of Behavioral Nutrition and Physical Activity 7, 88 (2010).
58. Mitchell, J. A., Pate, R. R., Beets, M. W. & Nader, P. R. Time spent in sedentary behavior and changes in childhood BMI: a longitudinal study from ages 9 to 15 years. Int J Obes 37, 54–60 (2013).
59. Keane, E. et al. Physical Activity, Sedentary Behavior and the Risk of Overweight and Obesity in School-Aged Children. Pediatr Exerc Sci 29, 408–418 (2017).
60. Goldfield, G. S. et al. Effects of Modifying Physical Activity and Sedentary Behavior on Psychosocial Adjustment in Overweight/Obese Children. J Pediatr Psychol 32, 783–793 (2007).
61. Saunders, T. J. et al. Associations of Sedentary Behavior, Sedentary Bouts and Breaks in Sedentary Time with Cardiometabolic Risk in Children with a Family History of Obesity. PLoS One 8, e79143 (2013).
62. Gaya, A. R. et al. Association between time spent in sedentary, moderate to vigorous physical activity, body mass index, cardiorespiratory fitness and blood pressure. Ann Hum Biol 36, 379–387 (2009).
63. Ochoa, M. C., Moreno-Aliaga, M. J., Martínez-González, M. A., Martínez, J. A. & Marti, A. Predictor factors for childhood obesity in a Spanish case-control study. Nutrition 23, 379–384 (2007).
64. Treuth, M. S. et al. A Longitudinal Study of Sedentary Behavior and Overweight in Adolescent Girls. Obesity 17, 1003–1008 (2009).
65. Nogueira, J. A. D. & da Costa, T. H. M. Gender Differences in Physical Activity, Sedentary Behavior, and Their Relation to Body Composition in Active Brazilian Adolescents. J Phys Act Health 6, 93–98 (2009).
66. Sardinha, L. B. et al. Objectively Measured Time Spent Sedentary Is Associated With Insulin Resistance Independent of Overall and Central Body Fat in 9- to 10-Year-Old Portuguese Children. Diabetes Care 31, 569–575 (2008).
67. Epstein, L. H. et al. A Randomized Trial of the Effects of Reducing Television Viewing and Computer Use on Body Mass Index in Young Children. Arch Pediatr Adolesc Med 162, 239 (2008).
68. Gortmaker, S. L. et al. Reducing Obesity via a School-Based Interdisciplinary Intervention Among Youth. Arch Pediatr Adolesc Med 153, 409 (1999).
69. Robinson, T. N. Reducing Children's Television Viewing to Prevent Obesity. JAMA 282, 1561 (1999).
70. Burke, V. et al. Television, computer use, physical activity, diet and fatness in Australian adolescents. International Journal of Pediatric Obesity 1, 248–255 (2006).
71. Berkey, C. S., Rockett, H. R. H., Gillman, M. W. & Colditz, G. A. One-Year Changes in Activity and in Inactivity Among 10- to 15-Year-Old Boys and Girls: Relationship to Change in Body Mass Index. Pediatrics 111, 836–843 (2003).
72. Fulton, J. E. et al. Physical Activity, Energy Intake, Sedentary Behavior, and Adiposity in Youth. Am J Prev Med 37, S40–S49 (2009).

73. Berkey, C. S. et al. Activity, Dietary Intake, and Weight Changes in a Longitudinal Study of Preadolescent and Adolescent Boys and Girls. Pediatrics 105, e56–e56 (2000).
74. Steffen, L. M., Dai, S., Fulton, J. E. & Labarthe, D. R. Overweight in Children and Adolescents Associated with TV Viewing and Parental Weight. Am J Prev Med 37, S50–S55 (2009).
75. Pratt, C. et al. Sedentary Activity and Body Composition of Middle School Girls. Res Q Exerc Sport 79, 458–467 (2008).
76. Laurson, K. R. et al. Combined Influence of Physical Activity and Screen Time Recommendations on Childhood Overweight. J Pediatr 153, 209–214 (2008).
77. BUTTE, N. F., PUYAU, M. R., ADOLPH, A. L., VOHRA, F. A. & ZAKERI, I. Physical Activity in Nonoverweight and Overweight Hispanic Children and Adolescents. Med Sci Sports Exerc 39, 1257–1266 (2007).
78. Ortega, F. B. et al. Cardiorespiratory Fitness and Sedentary Activities Are Associated with Adiposity in Adolescents. Obesity 15, 1589–1599 (2007).
79. Obarzanek, E. et al. Energy intake and physical activity in relation to indexes of body fat: the National Heart, Lung, and Blood Institute Growth and Health Study. Am J Clin Nutr 60, 15–22 (1994).
80. Katzmarzyk, P. T., Malina, R. M., Song, T. M. K. & Bouchard, C. Television viewing, physical activity, and health-related fitness of youth in the Québec family study. Journal of Adolescent Health 23, 318–325 (1998).
81. Goldfield, G. S. et al. Effects of Open-Loop Feedback on Physical Activity and Television Viewing in Overweight and Obese Children: A Randomized, Controlled Trial. Pediatrics 118, e157–e166 (2006).
82. Oliveira, L. C., Ferrari, G. L. de M., Araújo, T. L. & Matsudo, V. Overweight, obesity, steps, and moderate to vigorous physical activity in children. Rev Saude Publica 51, (2017).
83. Johansson, E. et al. Objectively measured physical activity in two-year-old children – levels, patterns and correlates. International Journal of Behavioral Nutrition and Physical Activity 12, 3 (2015).
84. Riddoch, C. J. et al. Objective measurement of levels and patterns of physical activity. Arch Dis Child 92, 963–969 (2007).
85. COLLINGS, P. J. et al. Physical Activity, Sedentary Time, and Fatness in a Biethnic Sample of Young Children. Med Sci Sports Exerc 49, 930–938 (2017).
86. Tambalis, K. D., Panagiotakos, D. B., Psarra, G. & Sidossis, L. S. Concomitant Associations between Lifestyle Characteristics and Physical Activity Status in Children and Adolescents. J Res Health Sci 19, e00439 (2019).
87. van Sluijs, E. M. F. et al. School-level correlates of physical activity intensity in 10-year-old children. International Journal of Pediatric Obesity 6, e574–e581 (2011).
88. Henderson, M. et al. How Are Physical Activity, Fitness, and Sedentary Behavior Associated With Insulin Sensitivity in Children? Diabetes Care 35, 1272–1278 (2012).
89. Al-Hazzaa, H. M. & Al-Rasheedi, A. A. Adiposity and Physical Activity Levels among Preschool Children in Jeddah, Saudi Arabia. www.smj.org.sa.

90. Leppänen, M. H. et al. Physical activity intensity, sedentary behavior, body composition and physical fitness in 4-year-old children: results from the ministop trial. Int J Obes 40, 1126–1133 (2016).
91. Collings, P. J. et al. Levels and patterns of objectively-measured physical activity volume and intensity distribution in UK adolescents: the ROOTS study. International Journal of Behavioral Nutrition and Physical Activity 11, 23 (2014).
92. Wang, C., Chen, P. & Zhuang, J. A National Survey of Physical Activity and Sedentary Behavior of Chinese City Children and Youth Using Accelerometers. Res Q Exerc Sport 84, S12–S28 (2013).
93. McVeigh, J. A., Norris, S. A., Cameron, N. & Pettifor, J. M. Associations between physical activity and bone mass in black and white South African children at age 9 yr. J Appl Physiol 97, 1006–1012 (2004).
94. Ojiambo, R. M. et al. Effect of Urbanization on Objectively Measured Physical Activity Levels, Sedentary Time, and Indices of Adiposity in Kenyan Adolescents. J Phys Act Health 9, 115–123 (2012).
95. Prista, A. et al. Physical Activity Assessed by Accelerometry in Rural African School-Age Children and Adolescents. Pediatr Exerc Sci 21, 384–399 (2009).
96. Ni Mhurchu, C. et al. Couch potatoes to jumping beans: A pilot study of the effect of active video games on physical activity in children. International Journal of Behavioral Nutrition and Physical Activity 5, 8 (2008).
97. Taylor, R. W. et al. School playgrounds and physical activity policies as predictors of school and home time activity. International Journal of Behavioral Nutrition and Physical Activity 8, 38 (2011).
98. Hume, C. et al. Does Weight Status Influence Associations Between Children's Fundamental Movement Skills and Physical Activity? Res Q Exerc Sport 79, 158–165 (2008).
99. Maddison, R., Foley, L. S., Olds, T. S., Ridley, K. & Jiang, Y. Validating the multimedia activity recall for children and adolescents in a large New Zealand sample. J Sports Sci 32, 470–478 (2014).
100. Fraysse, F., Grobler, A. C., Muller, J., Wake, M. & Olds, T. Physical activity and sedentary activity: population epidemiology and concordance in Australian children aged 11–12 years and their parents. BMJ Open 9, 136–146 (2019).
